# A Computational Neural Network Model for College English Grammar Correction

**DOI:** 10.1155/2022/9592200

**Published:** 2022-09-05

**Authors:** Xingjie Wu

**Affiliations:** School of General Caliber-oriented Education, Wuchang University of Technology, Wuhan 430000, China

## Abstract

For the error correction of English grammar, if there are errors in the semantic units (words and sentences), it will inevitably affect the subsequent text analysis and semantic understanding, and ultimately reduce the overall performance of the practical application system. Therefore, intelligent error detection and correction of the word and grammatical errors in English texts is one of the key and difficult points of natural language processing. This exploration innovatively combines a computational neural model with college grammar error correction to improve the accuracy of college grammar error correction. It studies the computational neural model in English grammar error correction based on a neural network named Knowledge and Neural machine translation powered College English Grammar Typo Correction (KNGTC). First, the Recurrent Neural Network is introduced, and the overall structure of the English grammatical error correction neural model is constructed. Moreover, the supervised training of Attention is discussed, and the experimental environment and experimental data are given. The results show that KNGTC has high accuracy in college English grammar correction, and the accuracy of this model in CET-4 and CET-6 writing can reach 82.69%. The English grammar error correction model based on the computational neural network has perfect function and strong error correction ability. The optimization and perfection of the model can improve students' English grammar level, which has certain practical value. After years of continuous optimization and improvement, English grammar error correction technology has entered a performance bottleneck. This mode's construction can break the current technology's limitations and bring a better user experience. Therefore, it is very valuable to study the error correction model of English grammar in practical application.

## 1. Introduction

Language processing technology is the product of the development and evolution of computer technology. It can enable computers to correctly understand and use natural language. It is a theoretical basis and method to realize the efficient communication between humans and computers. Specifically, to communicate in natural language is to enable computers to understand the ideas and meanings people convey in natural language, and to convey certain intentions and strategies in the written form of natural language. The first is to understand natural language, and the second is to generate natural language [1]. English is the most widely used international language, and writing is a crucial indicator of English level. Automatic check and correction of spelling errors of words and grammatical errors of sentences through computer natural language processing technology will be very conducive to improving students' English writing level [2]. Moreover, it also greatly saves the time and energy for teachers to review students' compositions, so that teachers can focus on the composition's overall structure and the narration content [3]. With the progress of automatic scoring technology for English essays, spelling and grammatical errors will become indispensable evaluation indicators, improving the objectivity and accuracy of automatic marking and scoring [4].

Experts and scholars have extensively researched grammatical errors in English sentences. Boyd's research showed that the ICICLE system detected grammatical abnormalities in sentences with the assistance of the constructed grammatical error recognition rules, and gave relevant prompt information [4]. Coley et al. checked grammatical errors through the *N*-ary grammar model, established a list of the candidate recommended words, and prioritized the candidate words according to the random context-free grammar [5]. Fetaya et al. focused on the preposition errors. With the British National Corpus (BNC) corpus as the training set, they extracted the context of prepositions, then established feature vectors and applied them to the generated maximum entropy model for preposition error detection. Among the contextual features of prepositions, the contextual word features in the extraction window contribute more than collocation features and named entities [6]. Domestic Gan et al. proposed a method based on examples and the introduction of negative rules to check the syntax. It is found that among the sentences with grammatical errors, 55% belong to local grammatical errors and 18% belong to global grammatical errors [7]. To sum up, in the extraction of context features, different features have different contributions to grammar checking. If the allocation of features is unreasonable, the discrimination of features will decline and ultimately affect the comprehensive performance of the system, so it is crucial to build a reasonable feature validity allocation function.

First, the research background of college English grammatical error correction is analyzed. It is found that college English grammar error correction has the characteristics of limited information, various error types and relatively single alignment. According to its characteristics, a spelling error correction model based on a neural network is generated based on the Sq2seg network. The transition probability between keys is obtained through the analysis and processing of user data, and the standard Attention matrix is generated according to the transition probability of keys. The supervised training of the neural network Attention mechanism is realized by adding regular terms to the neural network loss function. Finally, the above model is verified by experiments.

The English grammar correction model established adopts the idea of using the computer to replace the manual operation, so that teachers can get rid of the heavy task of English homework correction. In addition, from the students' point of view, these correction suggestions can also help students improve their English writing ability without teachers' guidance.

## 2. Materials and Methods

### 2.1. Recurrent Neural Network (RNN)

The neural network is like a simulation of the brain in processing information. Just like when people listen to a song or read a text, they must read information from front to back [8]. In traditional neural networks, each input is independent. However, in speech and natural language, the subsequent input is often affected by the previous input, which is called sequence information [9]. For example, when the next word in a sentence is predicted, the model must calculate according to the previously input word. RNN is a neural network for processing timing information, which is specially used to process timing information [10]. One of the main features of RNN is parameter sharing, which means there are many basic units in the RNN. These basic units have the same parameters, complete the same work, and transmit the information received by the model on different bases by transmitting the state of the hidden layer [11]. Therefore, at any time point, the model can obtain the input information of the current time, and take the previous input information into account. The characteristics of RNN make it especially suitable for processing natural language data [12].

**Figure 1:**
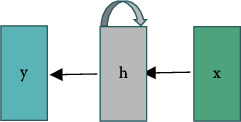
Folded view of RNN model structure.

The RNN basic unit consists of three main modules [13].  Figure 1 shows the basic structure:

1. Input layer *X* = {*x*_0_, *x*_1,_ …, x_*t*−1_, *x*_*t*_}
2. Output layer *Y* = {*y*_0_, *y*_1_,…, y_*t*−1_, *y*_*t*_}
3. Hidden layer *H* = {*H*_0_, *H*_1_,…, *H*_*t*−1_, *H*_*t*_}

**Figure 2:**
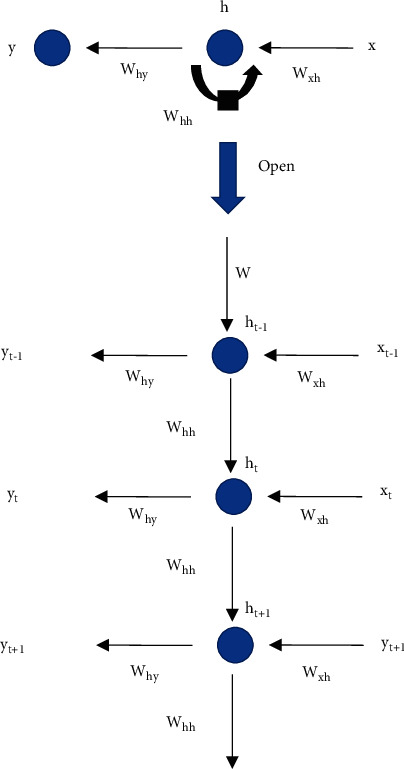
Expanded view of RNN model structure.

The RNN folded structure diagram in Figure 1 is expanded into the structure diagram in Figure 2:

Figure 2 shows that due to the structural characteristics of RNN, the parameters are mainly divided into three parts [14–16]:

1. Connection weight *W*_xh_ from the input layer to the hidden layer
2. Connection weight *W*_hh_ between hidden layers
3. Connection weight *W*_hy_ from the hidden layer to the output layer

The main purpose of the RNN model structure is to process and predict sequence data. In the fully connected neural network or convolutional neural network model introduced before, the network structure is from the input layer to the hidden layer and then to the output layer. The layers are fully connected or partially connected, but the nodes between each layer are not connected. RNN network has been proved to perform well in multiple natural language processing tasks. Due to the parameter sharing mechanism, the RNN network can greatly reduce the number of parameters in the model [17, 18]. It suggests that the parameters *W*_xh_, *W*_hh_ and *W*_hy_ are the same for each time step.

**Figure 3:**
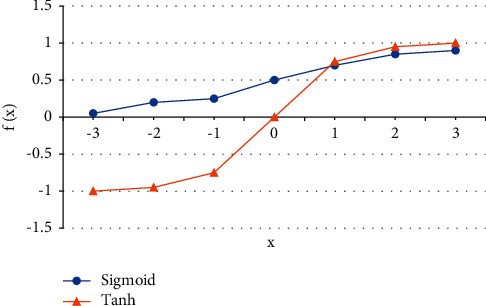
Common activation functions in RNN.

RNN also has some limitations. In the case of a deep network, due to the chain derivation rule, the updated value of parameters is often the result of the objective function multiplied by the gradient of several activation functions [19].  Figure 3 shows the commonly used activation functions in RNN networks:

Figure 3 suggests that the gradient of the activation function commonly used in neural networks is usually a value less than 1. The updated value of the parameters in front of the model will be multiplied by several activation function gradients, which will cause the updated value of the parameters to become smaller and smaller until it becomes 0, so that the parameters in front of the model cannot be effectively updated. This problem is called the gradient vanishing problem, also known as the long-distance dependence problem [20]. The gradient vanishing problem leads to even if the time series processed by the RNN network can be of any length in theory, with the deepening of the model, the parameter update rate of the former hidden layer of the model is far lower than that of the latter hidden layer of the model, and the former input of the model is difficult to affect the latter input [21].

### 2.2. The Overall Framework of the Computational Neural Model for College English Grammar Correction

Currently, the key problems of college English grammar error correction are mainly as follows. First, the abbreviations and irregular word orders of entities (names of people and places) in English texts will affect the accuracy of computer clauses. Second, in the context of feature extraction, different features have different contributions to grammar checking. If the allocation of features is unreasonable, the discrimination of features will decline and ultimately affect the system's overall performance. Third, the current research on grammar checking mainly focuses on the analysis of some types of grammatical errors, while the adaptability to other types of errors is not good enough. A Knowledge and Neural machine translation powered College English grammar Typo Correction (KNGTC) model is proposed for the problems in the existing methods. Neural Machine Translation (NMT) refers to a machine translation method that directly uses neural networks to carry out translation modeling in an end-to-end manner [22]. The error correction model based on NMT adopts a simple and intuitive method to complete the error correction, which has the following advantages. First, the process of input segmentation and processing one by one is omitted to avoid the uncertainty caused by segmentation errors. Second, it can effectively grasp the global information of user input, better fit the distribution of user input habits through the neural network, and have stronger adaptability and error correction ability.

**Figure 4:**
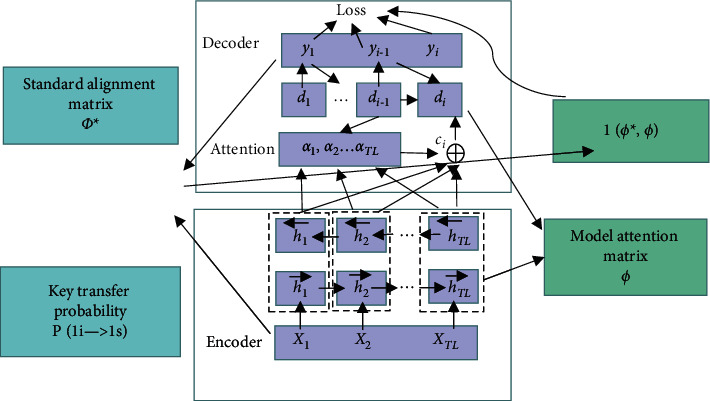
Overall network architecture of KNGTC.

College English grammar correction is regarded as a special translation task. It is determined that the input granularity of the model is character level and the output is statement level.  Figure 4 shows the main model structure of KNGTC:

In Figure 4, *X* = (*x*_1_, *x*_2_,…, *x*_*n*_) is adopted to express user input, where *x*_i_ represents a letter in input. *Y* = (*y*_1_, *y*_2_,…, *y*_Ts_) represents the result, and *y*_i_ represents a word. Error correction is conducted through the improved neural machine translation model. Besides, KNGTC can effectively improve error correction accuracy by combining the user's vector expression and the transition probability of adjacent keys.

KNGTC model is based on the RNN+ Attention structure in neural network machine translation. The Attention mechanism is to give a set of vector set values and a vector query. It is a mechanism that calculates the weighted sum of values based on the query. The focus of Attention is the calculation method of the “weight” of each value in the set values. Sometimes, this Attention mechanism is called query output, which focuses on (or takes into account) different parts of the original text. The RNN architecture is called the Seq2seq model. The Encoder part inputs a sequence *X* = (*x*_1_,…, x_TL_) as the source language, and the coding network generates a series of hidden layer states *H*= (*h*_1_,…, *h*_TL_) as the vector expression of the input through a bidirectional RNN. The hidden layer *h*_t_ is the result of splicing the hidden layer results of the positive RNN and the hidden layer results of the negative RNN at time *t*. The expression of H is as follows [23]:

(1) $$\begin{array}{c} h_{1}={\left[{\overset{⟶}{h_{t}}}^{T};{\overset{⟵}{h_{t}}}^{T}\right]}^{T}, \end{array}$$

$\overset{⟶}{h_{t}}$ and $\overset{⟵}{h_{t}}$ are calculated by the following equation [24]:

(2) $$\begin{array}{c} \overset{⟶}{h_{t}}={GRU}_{\overset{⟶}{enc}}\left(\overset{⟶}{h_{t-1}},x_{t}\right),\\ \overset{⟵}{h_{t}}={GRU}_{\overset{⟵}{enc}}\left(\overset{⟶}{h_{t+1}},x_{t},x_{t}\right), \end{array}$$

where ${GRU}_{\overset{⟶}{enc}}$ and ${GRU}_{\overset{⟵}{enc}}$ represent the forward and reverse gated recurrent unit (GRU), and different parameters are used respectively.

In the Decoder phase, the model still uses GRU units. The hidden layer state in the decoding network is called *d* = {*d*_1_, *d*_2_,…, *d*_Ty_}, and the probability distribution information of the current output sequence *y*_1_, *y*_2_,…, y_Ty_ is as follows [25]:

(3) $$\begin{array}{c} p\left(y_{i}|y_{1},\ldots ,y_{i-1},X\right)=g\left(y_{i-1},d_{i},c_{i}\right), \end{array}$$

*g* is a function of the nonlinear multi-layer structure. The user calculates the probability distribution information of output *y*_*i*_. *d*_*i*_ is the hidden layer state of RNN at the time *i*, and its equation is as follows:

(4) $$\begin{array}{c} d_{i}={GRU}_{dec}\left(d_{i-1},y_{i-1},c_{i}\right). \end{array}$$

The context information *c*_i_ used to predict *y*_*i*_ is equal to ∑_*k*=1_^*T*_*x*_^exp   (*q*_*ij*_). The weight *a*_*ij*_ of the hidden layer state *h*_*i*_ of the coding network is calculated by the following equation [26]:

(5) $$\begin{array}{c} a_{ij}=\frac{\exp\;\left(q_{ij}\right)}{\sum_{k=1}^{T_{x}}\exp\;\left(q_{ij}\right)}, \end{array}$$

where *q*_*ij*_ = *a* (*d*_*i*−1_, *h*_*j*_), *a* is a similarity calculation equation with back-propagation property, which updates the parameters together with the global network. The size of training data *C* is |*C*| and each training data is composed of the form of (*x*, *y*). The loss function of the model is as follows:

(6) $$\begin{array}{c} Loss^{\prime }=-\sum_{\langle x,y\rangle \in ∁}\sum_{l=1}^{T_{y}}logp\left(y_{l}|y_{<l},x\right). \end{array}$$

### 2.3. Supervised Training of Attention Mechanism

The supervised training of the Attention mechanism is a machine learning task of inferring functions from labeled training datasets. The training data consists of a set of training examples. In supervised learning, each example is a pair consisting of an input object (usually a vector) and the desired output value (also known as a supervised signal). A supervised training algorithm analyzes training data and generates an inference function, which can be used to map new examples. The purpose of supervised training of the Attention mechanism is to carry out supervised training of the Attention mechanism in combination with key transfer probability and input-output alignment information. This method can effectively correct click errors and the simplified spelling in the Pinyin input method.

Attention weights *α*_1_, *α*_2_,…, and *α*_TL_ play an important role in predicting the next output in decoding networks. However, in the traditional Attention mechanism, only the information of the source language itself is considered, and other information is not effectively used. In the English grammar error correction task, a multi-level Attention mechanism is introduced to improve the error correction rate of the model at the word level and character level. In this project, alignment information and adjacent key transfer probability greatly impact spelling error correction tasks. Therefore, this section describes how to introduce alignment information and adjacent key transfer probability into the Attention mechanism as external information. A new alignment model is proposed to construct a binary matrix representing the input-output alignment relationship, and it is initialized with “0.” Then, the key transfer probability is adopted to fill in the aligned position, and the supervised training of the Attention mechanism is realized through this matrix [27].

For each training data, the standard alignment matrix automatically generated by the model is defined as middle *ϕ*^*∗*^, as shown in:

(7) $$\begin{array}{c} \begin{array}{c} \begin{array}{cccc} s_{1} & s_{2} & s_{3} & eos \end{array}\\ \begin{array}{c} l_{1}\\ l_{2}\\ l_{3}\\ l_{4}\\ l_{5}\\ eos \end{array}\left[\begin{array}{cccc} 0.93 & 0 & 0 & 0\\ 0.57 & 0 & 0 & 0\\ 0 & 0.97 & 0 & 0\\ 0 & 0.49 & 0 & 0\\ 0 & 0 & 0.86 & 0\\ 0 & 0 & 0 & 1 \end{array}\right], \end{array}\\ \begin{array}{c} \begin{array}{cccc} s_{1} & s_{2} & s_{3} & eos \end{array}\\ \begin{array}{c} l_{1}\\ l_{2}\\ l_{3}\\ l_{4}\\ l_{5}\\ eos \end{array}\left[\begin{array}{cccc} 0.62 & 0 & 0 & 0\\ 0.38 & 0 & 0 & 0\\ 0 & 0.67 & 0 & 0\\ 0 & 0.33 & 0 & 0\\ 0 & 0 & 0.1 & 0\\ 0 & 0 & 0 & 1 \end{array}\right] \end{array}. \end{array}$$

The input of *φ*^*∗*^ is (*l*_1_,…, *l*_TL_), the corresponding number of lines is *T*_L_ + *l* (representing *T*_L_ inputs and one <eos>), and the output is (*S*_1_,…, *S*_TS_). The corresponding number of columns is *T*_s_ + *l* (*T*_s_ outputs plus one <eos>). For *l* < *i* < *T*_L_, *l* < *j* < *T*_s_, ∅_*ij*_^*∗*^ is as follows:

(8) $$\begin{array}{c} \emptyset_{ij}^{*}=\underset{1\leq k\leq \left|s_{j}\right|}{\max}p_{t}\left(S_{jk}⟶l_{i}\right)A_{i,j}, \end{array}$$

where *A*_*ij*_ indicates whether the character *l*_*i*_ is part of the spelling sentence *S*_*j*_. If yes, it is *l*. Otherwise, it is 0. *S*_*jk*_ represents the *k*-th character in the word *j*. For *i* = *T*_L_ + *l* or *j* = *T*_S_ + *l*:

(9) $$\begin{array}{c} \emptyset_{i,j}=\left\{\begin{array}{c} \begin{array}{cc} 1, & i=T_{L}+1,j=T_{S}+1,\\ 0, & otherwise. \end{array}\\ \; \end{array}\right. \end{array}$$

First, the input sequence *L* and the corresponding word sequence *S* are obtained. Recursively, it is essential to traverse all possible separated results of *L*. Then, each result is scored. The scoring basis is to separate each corresponding part of the result and *s*, take the editing distance and sum, and take the result of the minimum score as the most reasonable segmentation result of the input string *L*. For example, if a division result of *L* is Seg = {*seg*_1_, *seg*_2_,…, *seg*_*T*_*S*__}, and the CALCSCORE calculation equation in line 29 is [28]:

(10) $$\begin{array}{c} \tau =-\sum_{n=1}^{T_{S}}EditDistance\left({seg}_{n},s_{n}\right), \end{array}$$

where Edit Distance represents the editing distance, and refers to the minimum number of editing operations required to convert one string to the other between two strings. The greater their distance is, the more different they are. Editing operations include replacing one character with another, inserting characters, and deleting a character.

The traditional Attention matrix calculates the default alignment matrix *ϕ*^*∗*^ based on the hidden layer of the coding network. The matrix distance between *ϕ*^*∗*^ and *ϕ*' is calculated by the following equation [29]:

(11) $$\begin{array}{c} 1\left(\emptyset^{*},\emptyset \right)={\left\|\emptyset^{*}-\emptyset \right\|}_{2}^{2}. \end{array}$$

A new loss function is obtained by combining it with the original loss function:

(12) $$\begin{array}{c} Loss=-\sum_{\langle x,y\rangle >\in ∁}\left\{\sum_{l=1}^{T_{y}}\log\;p\left(y_{l}|y_{<1},x\right)+1\left(\emptyset^{*},\emptyset \right)\right\}. \end{array}$$

It reveals that the new loss function consists of two parts. The first part measures the accuracy of the error correction results, and the second part measures the accuracy of the alignment model.

### 2.4. Experimental Environment

**Figure 5:**
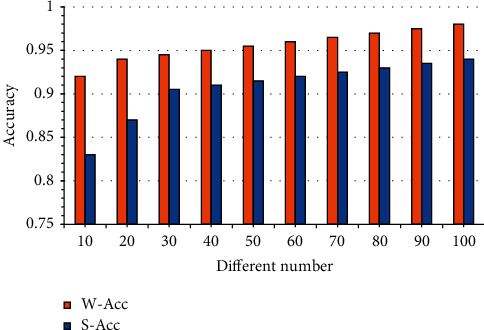
Accuracy of the model under the condition of taking the optimal results of different numbers.

The uniform distribution initializes the parameters in the experiment. Both the encoding and decoding networks use a 128 dimension hidden layer, and the dimension of the input layer is 256. The batch size is 64 samples, and the parameter optimization algorithm is Adam algorithm. The dropout method is used to prevent overfitting, and the dropout ratio is 0.2. KNGTC model has been trained on a Tesla K40 GPU for 48 hours, and has reached the convergence state after 350000 recursions. The decoding phase uses the Beam Search algorithm.  Figure 5 shows the model's accuracy under different decoding widths in the Beam Search algorithm.

Figure 5 shows that the model accuracy increases with the increase of decoding width. However, due to the response time requirement of the input method, too many candidate results will bring a lot of computation to the subsequent processing of the model. Therefore, in the decoding stage, the top ten results in the model output set is adopted to evaluate the model effect.

### 2.5. Experimental Dataset

The content used for testing in this section includes three parts: non-word error processing, true word error processing, and sentence grammar processing. Here, English texts with different degrees of difficulty are selected to test the impact of the selection of training corpus on the construction of dictionaries. The compositions of non-English majors (ST3 and ST4) in the Chinese learner corpus are taken as test examples of non-word error processing and sentence grammar processing. Four topics are selected, a total of 120 compositions (Table 1). These 120 compositions have non-lexical errors and grammatical errors in varying degrees, and have been manually marked with errors. Table 1 shows the specific symbol marking information related to the test. In addition, some common misspelled words are extracted from CET-4 and CET-6 exercises as supplementary test examples of non-word error handling. For the true word error test, the remaining 20% of the sentences related to the confusion set in the training corpus are taken as test examples.

## 3. Results and Discussion

### 3.1. Grammar Error Handling

In order to check and correct the grammatical errors of sentences, one of the most important problems is to deal with the text sentence segmentation. This system uses the rule-based sentence segmentation method, which adds a hypothetical boundary to the input text, and then uses the rule method to correct errors and realize the sentence segmentation function. The test examples come from 120 compositions in the experimental data.  Table 2 shows the specific punctuation distribution.

Table 2 shows that if the question mark, exclamation marks and periods are directly taken as sentence boundaries, the sentence segmentation accuracy is only 66.43%. The result of sentence segmentation is the basis of sentence grammatical error analysis, so it is necessary to correct the hypothetical sentence boundary. The following is the sentence segmentation results of the test case by the system using the method based on error correction rules (Table 3).

The above error correction results show that the sentence segmentation accuracy is 98.96%, and the error correction effect is obvious.

**Figure 6:**
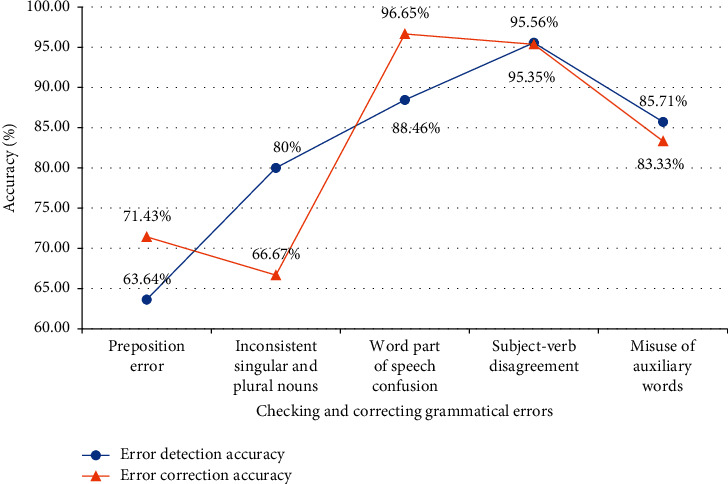
Grammar test results of the selected instance to be tested.

By studying and analyzing the grammatical errors in CET-4 and CET-6 composition, this system uses the combination of a neural network model and artificial grammar rules to solve the common grammatical errors in writing. 110 test cases with these grammatical errors are extracted from the experimental data text, and some contain more than one kind of grammatical error. In the test, the system's error detection and error correction accuracy for sentence grammatical errors are mainly investigated.  Figure 6 shows the test results.

The data in Figure 6 show that the average correction accuracy of the system for CET-4 and CET-6 compositions is 82.69%. Through the observation of the data, the accuracy of the system in detecting and correcting prepositional errors and the inconsistency of singular and plural nouns is not high enough. The reasons are explained below.

The number of prepositions involved in English is relatively large, and the context in which they are used is not fixed, which makes the weight distribution of context features extracted in the training process more scattered, and the discrimination of features is low. Besides, during the training process, some prepositions contain similar contextual features, such as prepositions “in” and “on.” Based on these two main reasons, the system is not easy to find the optimal preposition in prediction, which reduces the performance of error detection and correction.

The inconsistency between singular and plural nouns involves two cases: mistakenly writing uncountable nouns into plural form and mistakenly writing plural form of countable nouns into a singular form. Because some nouns can be treated as both countable and uncountable in actual writing, there will be some misjudgments and omissions. Besides, there are many kinds and numbers of leading words to identify whether the noun should be the plural form, and there are still some omissions in the definition of rules. It is believed that the system performance can be improved with the improvement of the rules.

### 3.2. KNGTC and Comparison Test Accuracy

**Figure 7:**
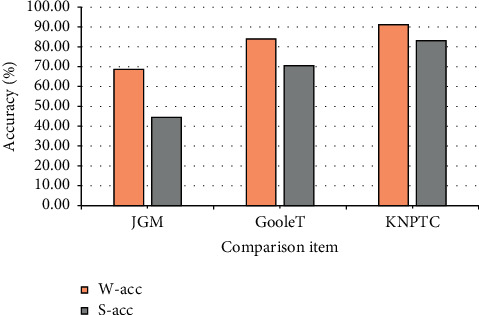
Accuracy of KNGTC and comparison models.

The KNGTC model is compared with two comparative models to evaluate its performance. First, the probabilistic graphical model (PGM). The method of PGM is used to realize the training of English grammar error correction. Their model finds a joint global optimal correction type in the whole input statement sequence. Second, Google translate (Google *T*). Google *T* is a translation software developed by Google, which supports grammar checking. To some extent, it can represent the performance of mature language translation technologies in the current market.  Figure 7 shows the accuracy comparison of the three modes:

Figure 7 shows that KNGTC outperforms the PGM model in almost all data types, especially on LAP and Google *T*. The main reason is that the translation information contains less information and is sparse. The PGM model can hardly deal with the user input in simplified form, while KNGTC can effectively generate reasonable semantic information and spelling form according to the global information. In addition, KNGTC can use key transfer probability and alignment information, so it can select more reasonable candidate results.

KNGTC performs as well as or even better than Google *T* in various data types, and achieves an accuracy improvement of nearly 17% in the comprehensive results. After analysis, it is found that the main reason for the poor performance of Google *T* is that the existence of simplified spelling or wrong input affects its accurate separation of user input. However, KNGTC overcomes this problem.

## 4. Conclusion

This work mainly studies the computational neural model of college English grammatical error correction. The main conclusions are as follows. (1) This work mainly summarizes RNN, establishes the overall architecture of the computational neural model of college English grammatical error correction, and studies the supervised training of Attention mechanism, experimental environment and dataset. (2) Through comparative experiments, it is found that the accuracy of grammar correction of KNGTC is higher than that of other models. The average accuracy of this model is 82.69%.

Due to the limited time and level, this work still has the following shortcomings. (1) The model can use a larger scale of corpus for training. The neural network model has a strong ability to fit data distribution. Theoretically, using more abundant user data can effectively enhance the error correction ability of the grammar error correction model. (2) The user's click position information is introduced into the model, and the input vector splicing method is used in the process. More ways can be considered to combine the prior information with the error correction model. (3) The reordering model of prediction results based on the language model is not deep enough, and the structure is not complex enough, which leads to less obvious improvement of the prediction effect.

## Figures and Tables

**Table 1:** Main data sources of grammatically incorrect texts.

Composition topic	Source	Quantity
Practice makes perfect	ST3	30
Global shortage of fresh water	ST3	30
My view on job-hopping	ST4	30
My view on fake commodities	ST4	30

**Table 2:** The punctuation of the text to be separated.

	Number of question marks	Number of exclamation marks	Number of periods	The actual number of sentences	Sentence boundary ratio (%)
Test case	531	11	1475	1340	66.43

**Table 3:** Sentence segmentation results.

	Number of correctly found sentences	Number of all sentences found	Sentence segmentation accuracy (%)
Test case	1345	1359	98.96

## Data Availability

The dataset used in this paper are available from the corresponding author upon request.
